# Supplementary material for: Predictive models for the selection of thermally tolerant corals based on offspring survival
Source: Nat Commun. 2022 Mar 29;13:1543. doi: 10.1038/s41467-022-28956-8 (PMC8964693; doi:10.1038/s41467-022-28956-8)
Supplement: Supplementary file 1 — Supplementary Information [file 41467_2022_28956_MOESM1_ESM.pdf]

Supplementary Material

Predictive models for the selection of thermally tolerant corals based on offspring survival
K. M. Quigley, M. J. H. van Oppen

Supplementary Results

Juvenile survival. Survival at 27°C and elevated temperatures varied both within and between the 25 crosses of larvae (Fig. 2a, Supplementary Table 1, Supplementary Fig. 3) and juveniles infected with the four Symbiodiniaceae treatments (Fig. 2b, Supplementary Fig. 4). Specifically, within each of the families and treatments, there was no significant difference in survival between the two temperature treatments for juveniles placed in the sediment, D1a, SS1, and C1 treatments (Supplementary Fig. 4; Wilcoxon test Bonferroni *P.adj* = all 1).

The lack of significant differences in survival was likely due to high variability in survival at both temperatures (Fig. 2). Elevated mortality of juveniles, even at 27°C conditions is common in corals and other organisms with mass-spawning reproductive strategies. For example, there is a well-documented drop in survival in the first few months of juvenile life, generally accounting for around >30-99% mortality (reviewed in [1]). Hence, we propose that this mortality at 27°C temperatures is expected, especially given the long timeframe of this experiment (58 days) covering this early window of juvenile life. Survival at both temperatures were incorporated into the Boosted Regression Tree models, allowing the machine learning algorithm to learn from patterns in survival of both 27°C and elevated temperature datasets and thereby incorporates this variability.

Surviving coral juveniles after eight weeks of heat stress at an elevated temperature of 32°C were exposed to a maximum of 24.7 DHW (Supplementary Fig. 6). This is equivalent to 8.7 additional DHW that were experienced by surviving corals in the 2016 mass bleaching (max 16 DHW [2]). 8.7 DHW is equivalent to 2.85 – 3.4 DHW above each of the reefs maximum summer temperatures during this experiment by about 2.6 - 3.08°C (hotspots). Therefore, surviving juveniles were able to withstand on average an increase of 2.9°C ± 0.08.

To verify previous Wilcoxon results and calculate the variance due to fixed and random effects, a generalized linear mixed effects model was run using a negative binomial distribution in lme4 using post-hoc two-sided Tukey tests (Supplementary Tables 4-5), and corroborated previous results.

Comparison of survival between larvae and juveniles. To assess if more thermally tolerant larvae grew into more thermally tolerant juveniles, survival at both life-stages was compared at the final timepoint for each life-stage (Supplementary Fig. 8). It should be noted that these life-stages were measured for different lengths of time, under different symbiont states (symbiotic and aposymbiotic) and at different elevated temperature treatments, so comparisons as such should be taken with caution. At 27°C, only cross CUxBK differed significantly in survival, in which larvae survived better than their juvenile counterparts (Wilcoxon test Bonferroni *P.adj* = 0.019; Supplementary Fig. 8).

In the elevated temperature treatments (35.5 or 32°C for larvae and juveniles, respectively), most of the crosses survived better as juveniles compared to as larvae (5 of the 22 crosses, ~22.7%, potentially due to a lower temperature), including juveniles from crosses, DRxDR

(Wilcoxon test Bonferroni *P.adj* = 0.038), LSxCU and LSxLS (*P.adj* = 0.0058 – 0.041). Only 1 of the 22 (4.5%) cross comparisons resulted in larvae that survived better compared to juveniles, including BKxSB and CUxBk (*P.adj* = 0.027 – 0.046) (Supplementary Fig. 5).

Environmental predictors. Seven environmental predictors were calculated from remotely sensed data and are further described in Supplementary Table 2. Briefly, these predictors are Daily Temperature Range (DTR), Latitude (LAT), Standard Deviation of the daily mean yearly temperature (SST_stdev), Frequency of standard deviation of SST anomalies (SSTA_Freq_stdev), mean yearly SST (SST_av.post), mean yearly SST pre-mass bleaching in 2016 (SST_av.pre), and the Standard Deviation of thermal stress anomaly (TSA_DHW_stdev).

Non-parametric Wilcoxon tests were used to determine if there were statistically significant differences in the mean value of predictors (Fig. 1c-h) across each reef (pairwise comparisons, $P < 0.05$ denoted by asterisks in Fig. 1c-h). SST_av.pre varied significantly between Curd and Sand Bank 7 (Fig. 1c; $P = 0.049$), Davies ($P = 3.6e-7$), and Backnumbers ($P = 1.5e-5$). SST_av.pre did not vary between Long Sandy and Curd or Sand Bank 7 ($P = 0.39$ and 0.17) but it did vary with Davies and Backnumbers ($P = 0.001$ and 7.4e-5). Sand Bank 7 also varied with Davies and Backnumbers ($P = 0.0027$ and 0.023). Davies and Backnumbers did not differ significantly in SST_av.pre ($P = 0.34$). SST_stdev did not differ significantly across any reef comparisons (Fig. 1d; non-parametric Wilcoxon test all $P = 0.38$ – 1). SST_av.post differed significantly between Curd reef and Long Sandy ($P = 0.02$), Sand Bank 7, ($P = 0.0.31$), Davies ($P = 0.0051$) and Backnumbers ($P = 0.02$), but not between any of the other reef combinations (Fig. 1e). SSTA_Freq_stdev differed significantly between Backnumbers and Curd ($P = 0.038$), Davies ($P = 8e-4$), Sand Bank 7 ($P = 0.055$), Long Sandy ($P = 0.032$) and Davies compared to Long Sandy ($P = 0.051$, Fig. 1g). TSA_DHW_stdev did not vary significantly between any reef combinations ($P = 0.087$ – 1, Fig. 1h).

Environmental predictors of heritable heat tolerance. TSA_DHW_stdev was the most influential factor in predicting aposymbiotic purebred larval survival under heat stress (32.2%), followed by SST_av.pre (29.1%) and SST_stdev (20.4%) (Fig. 3b). Alternatively, DTR, one of the key metrics in predicting adult coral responses to bleaching, was the most important predictor in aposymbiotic hybrid larval survival (69.9%).
In symbiotic juvenile corals, the most influential predictors varied by symbiont exposure, although TSA_DHW_stdev and SST_av.pre were important across many purebred/hybrids and symbiont types (Fig. 3b), especially in C1 (purebreds- SST_av.pre: 63.4%, hybrids- TSA_DHW_stdev: 54.7%) and D1a infected juveniles (purebreds- SST_av.pre: 58.1%, hybrids- TSA_DHW_stdev: 80.5%). Juveniles infected with SS1 were best explained by SSTA_Freq_stdev (purebreds: 40.4%) and TSA_DHW_stdev (45.9%). Survival under heat stress in juveniles exposed to northern "hot" sediments were best explained by SST_stdev (purebreds: 72.0%) and TSA_DHW_stdev (hybrids: 84.1%).

Predictive intrinsic resistance models. Although GBM models did relatively well at predicting juvenile survival when infected with C1 (R^2 = 10-27%, Fig. 3a), the overall survival of the C1 treatment at elevated temperature was lower than other treatments (Fig. 2). SS1 and stress-tolerant D1a provided an almost equivalent heat protection to juveniles at 32°C, although the explanatory power of the SS1 GBM models was poor (R^2 = 0.04 - 1, Fig. 3a). Hence, the treatments with the highest juvenile survival under heat stress and greatest

predictive power were the D1a and hot sediments treatments. The D1a treatment was chosen for further predictive modelling given improved predictive power and survival at ambient temperatures.

For all relevant environmental predictors, GBM model prediction partial plots were fit, allowing for the calculation of the intrinsic resistance models. Survival (%) of larvae and juveniles were estimated by plotting GBM model results (Fig. 3b) into standard equations (Fig. 4d-g insets). Given the importance of TSA_DHW_stdev in predicting purebred larval survival (32.2%, Fig. 4a), a polynomial predictive model eq. (1) was fit ($R^2 = 0.97$).

$$y = -23907x^3 + 87017x^2 - 105563x + 42719 \quad \textbf{(1)}$$

Following this, purebred larvae were predicted to have a 90.4 – 95.5% survival under heat stress should have parents sourced from locations that exhibit TSA_DHW_stdev of 1.08 (peach coloured sites, Fig. 5). Reef sites that are predicted to yield adults that will produce larvae of high survivorship could be found throughout the GBR, predominately on offshore reefs.

Hybrid larval survival under heat was best predicted by DTR (69.9%, Fig. 4b). The 5th order polynomial model fit to GBM predictions ($R^2 = 0.96$) followed eq. (2) to estimate DTR that resulted in optimal survival for hybrid larvae (~93.1% survival) at 0.515 DTR:

$$y = 3,229.762899734070000x^5 - 13,291.025769647700000x^4 + 21,697.985121632700000x^3 - 17,516.412067116400000x^2 + 6,900.397827213720000x - 960.624993280386000 \quad \textbf{(2)}$$

The sourcing of adults to produce hybrid larvae of high survival should be collected from sites along the GBR exhibiting DTR temperatures of ~0.515°C (rose coloured sites, Figs. 4b, 5). Similarly, to purebred larvae, these sites stretched across the GBR, but were particularly concentrated in offshore areas of the southern GBR.

Purebred juvenile survival at 32°C, when infected with D1a, was predominantly explained by SST_av.pre (58.1%, Figs. 4c). The 3rd order polynomial (R^2=0.97), followed eq. (3) to estimate which SST_av.pre temperature explained optimal survival of purebred juveniles under heat stress at 26.69°C.

$$y = -225.10864869249x^3+18334.5412796288x^2-497759.710251179x+4504526.46958609 \quad \textbf{(3)}$$

Gravid adult corals that would produce purebred juveniles of high survival should be collected from environments of SST_av.pre of 26.69°C (purple, Fig. 5), mainly around four sites of the central, offshore GBR.

Survival at heat of hybrid juveniles infected with D1a was predominantly explained by TSA_DHW_stdev (80.5%, Fig. 4d). The 3rd order polynomial model ($R^2 = 0.99$) estimated the TSA_DHW_stdev temperature for optimal hybrid juvenile survival (99.4%).

$$y = 10704x^3 – 39748x^2 + 49287x – 20321 \quad \textbf{(4)}$$

Similarly, to larval results, the collection of gravid adult corals to produce heat resistant hybrid juveniles could be collected across the GBR from sites with TSA_DHW_stdev temperatures around 1.319 (blue, Fig. 5). Potential collection sites were particularly concentrated in the Far Northern GBR.

Amplicon sequencing and RNAseq of Symbiodiniaceae communities. Amplicon ITS2 sequencing of single juveniles from the sediment treatment (n = 30 juveniles) resulted in very high read counts (88,857- 504,222 reads per sample). Amplicon sequencing revealed a high diversity of Symbiodiniaceae taxa in the sediments (385 Amplicon Sequence Variants-ASVs)

in this juvenile treatment (Supplementary Fig. 1). Generalized linear models performed in DESeq2 using Benjamini-Hochberg multiple test corrections accounting for temperature and tank replicates identified four significantly differentially abundant ASVs between these treatments (as outlined in the main text, Supplementary Fig. 1).

After variance normalization, symbiont communities within the 27°C treatment where generally dominated by *Cladocopium* and *Fugacium*, whilst the juveniles sequenced from the elevated temperature treatment had more individual juveniles with *Durusdinium* and the hitherto uncharacterized clade "I" (Supplementary Fig. 1). A high diversity of Symbiodiniaceae taxa in coral juveniles has been previously reported, including the occurrence of *Fugacium* and "I", recovered from juvenile samples in the wild [3]. Moreover, the occurrence of these taxa has been detected through the use of two different pipelines (OTU variant calling via USEARCH clustering [4], adapted for Symbiodiniaceae specifically [5] using custom databases) as well as with ASV calling (DADA2 [6], adapted for Symbiodiniaceae [7]). RNAseq of n = 43 different juvenile samples confirmed the mixed assemblage of symbionts from these genera (Quigley and Strader, *in-review*). The detection of these additional taxa could also be due to symbiont cells being present in the coelenteron of the juvenile corals and may not necessarily be in symbiosis. Further work using methods such as in situ fluorescence hybridization (FISH) would be needed to confirm if these cells are within the coral cells. Therefore, given the detection of these taxa in coral juveniles of this species previously from other sources and using other methods, we suggest that these ASVs do not represent spurious variants or host contamination.

Amplicon sequencing for juveniles from the D1a, SS1, and C1 treatments were low, and so RNAseq of additional, independent juveniles were used to confirm the dominant communities within these treatments. Specifically, ITS2 sequencing of single juveniles from the SS1 treatment (n = 11 juveniles) resulted in very low read counts (3- 46 reads per sample), potentially due to non-specific primer binding and suggests that new ITS2 primers are needed for this lab-evolved strain. However, RNAseq confirmed dominant *Cladocopium* across a larger number of different juvenile samples in this treatment (n = 36), the genera to which SS1 belongs. *Durusdinium* ITS2 sequencing also resulted in low read numbers (1-6 reads) across n = 9 juveniles, however, RNAseq data of n = 38 different juveniles confirmed the dominance of *Durusdinium* in this treatment. Finally, RNAseq of n = 29 juveniles and amplicon sequencing of different juveniles (n = 12; 31 to 2278 reads per sample) confirmed the dominant abundance of *Cladocopium* in the *Cladocopium* treatment.

Supplementary Table 1. Total number of maternal and paternal colonies used per cross. Grey columns summarize which crosses were used in each experiment, including field outplanting as described [8].

<table>
<tr><th>Cross</th><th>Total maternal colonies</th><th>Total paternal colonies</th><th>Larvae</th><th>Juvenile</th><th>Field</th></tr>
<tr><td>BK x BK</td><td>2</td><td>2</td><td bgcolor="grey"></td><td bgcolor="grey"></td><td bgcolor="grey"></td></tr>
<tr><td>BK x CU</td><td>7</td><td>7</td><td bgcolor="grey"></td><td bgcolor="grey"></td><td bgcolor="grey"></td></tr>
<tr><td>BK x DR</td><td>4</td><td>3</td><td bgcolor="grey"></td><td bgcolor="grey"></td><td bgcolor="grey"></td></tr>
<tr><td>BK x LS</td><td>3</td><td>5</td><td bgcolor="grey"></td><td bgcolor="grey"></td><td bgcolor="grey"></td></tr>
<tr><td>BK x SB</td><td>7</td><td>5</td><td bgcolor="grey"></td><td bgcolor="grey"></td><td></td></tr>
<tr><td>CU x BK</td><td>7</td><td>8</td><td bgcolor="grey"></td><td bgcolor="grey"></td><td bgcolor="grey"></td></tr>
<tr><td>CU x CU</td><td>6</td><td>5</td><td bgcolor="grey"></td><td bgcolor="grey"></td><td bgcolor="grey"></td></tr>
<tr><td>CU x DR</td><td>2</td><td>3</td><td bgcolor="grey"></td><td bgcolor="grey"></td><td></td></tr>
<tr><td>CU x LS</td><td>2</td><td>3</td><td bgcolor="grey"></td><td bgcolor="grey"></td><td></td></tr>
<tr><td>CU x SB</td><td>2</td><td>5</td><td bgcolor="grey"></td><td bgcolor="grey"></td><td></td></tr>
<tr><td>DR x BK</td><td>3</td><td>4</td><td bgcolor="grey"></td><td bgcolor="grey"></td><td bgcolor="grey"></td></tr>
<tr><td>DR x CU</td><td>3</td><td>2</td><td bgcolor="grey"></td><td bgcolor="grey"></td><td bgcolor="grey"></td></tr>
<tr><td>DR x DR</td><td>3</td><td>3</td><td bgcolor="grey"></td><td bgcolor="grey"></td><td bgcolor="grey"></td></tr>
<tr><td>DR x LS</td><td>3</td><td>3</td><td bgcolor="grey"></td><td></td><td></td></tr>
<tr><td>DR x SB</td><td>3</td><td>3</td><td bgcolor="grey"></td><td bgcolor="grey"></td><td bgcolor="grey"></td></tr>
<tr><td>LS x BK</td><td>4</td><td>7</td><td bgcolor="grey"></td><td bgcolor="grey"></td><td bgcolor="grey"></td></tr>
<tr><td>LS x CU</td><td>3</td><td>2</td><td bgcolor="grey"></td><td bgcolor="grey"></td><td></td></tr>
<tr><td>LS x DR</td><td>3</td><td>3</td><td bgcolor="grey"></td><td></td><td></td></tr>
<tr><td>LS x LS</td><td>5</td><td>6</td><td bgcolor="grey"></td><td bgcolor="grey"></td><td bgcolor="grey"></td></tr>
<tr><td>LS x SB</td><td>6</td><td>8</td><td bgcolor="grey"></td><td bgcolor="grey"></td><td bgcolor="grey"></td></tr>
<tr><td>SB x BK</td><td>3</td><td>7</td><td bgcolor="grey"></td><td bgcolor="grey"></td><td></td></tr>
<tr><td>SB x CU</td><td>3</td><td>2</td><td bgcolor="grey"></td><td></td><td></td></tr>
<tr><td>SB x DR</td><td>3</td><td>3</td><td bgcolor="grey"></td><td bgcolor="grey"></td><td bgcolor="grey"></td></tr>
<tr><td>SB x LS</td><td>3</td><td>3</td><td bgcolor="grey"></td><td bgcolor="grey"></td><td bgcolor="grey"></td></tr>
<tr><td>SB x SB</td><td>5</td><td>6</td><td bgcolor="grey"></td><td bgcolor="grey"></td><td bgcolor="grey"></td></tr>
</table>

Supplementary Table 2. Sea Surface Temperature metrics derived from global (CoRTAD (*33*)) and modelled GBR-specific (eReefs (*34*)) remotely sensed data used in models. CoRTAD timeframe: 1982-01-02 to 2019-12-27.

Metric	Unit	Definition	Source
Lat	Decimal degrees	Latitude	NA
SST_av.post	°C	Average annual temperature averaged across daily recordings. Mean SST (full year) – including 2016, 2017 mass bleaching.	eReefs<br>01/01/2013 – 28/02/2018, 1km, daily
SST_av.pre	°C	Average annual temperature averaged across daily recordings. Mean SST (full year) – before mass bleaching.	eReefs<br>01/12/2014 – 31/10/2016, 1km
SST_stdev *sensu* (*10*)	°C	Standard deviation of the mean yearly temperature averaged across daily recordings.	eReefs<br>01/12/2014 – 31/10/2016, 1km
TSA_DHW_stdev *sensu* (*10*)	°C – weeks	Standard deviation of Thermal Stress Anomaly calculated in degree heating weeks. Cumulative Thermal Stress DHW_{4km}	CoRTAD 4km weekly, standard deviation of sum of previous 12 weeks when TSA is greater than or equal to 1°C
SSTA_Freq_stdev *sensu* (*10*)	°C – weeks	Frequency of standard deviation of SST anomalies.	CoRTAD 4km weekly, Standard deviation of number of times over previous 52 weeks that SSTA is greater than or equal to 1°C
DTR (Daily Temperature Range) *sensu* (*28*)	°C	High-frequency temperature variability.	eReefs<br>2013-2020,<br>1km

Supplementary Table 3. Total number of unique maternal and paternal colonies used per reef.

Reef	Total maternal colonies	Total paternal colonies
BK	7	11
DR	6	12
CU	8	16
LS	7	15
SB	7	14

Supplementary Table 4. Summary table of statistical results for generalized linear mixed effects models for juvenile survival probabilities comparing models with and without "cross" as a random effect (*P-adjusted* value = two-sided Tukey post-hoc tests).

Model	Response	Distribution	Link	Predictors	Variance	Std. Dev
1	Survival (%)	Negative binomial	log	Temperature * Symbiont treatment	0.00434174	0.06706009
				Tank (R)	0.006048	0.07777
				Cross (R)	0.049744	0.22303
2	Survival (%)	Negative binomial	log	Temperature * Symbiont treatment	0.004275838	0.0456636
				Tank (R)	0.005616	0.07494

Supplementary Table 5. Summary table of statistical results for generalized linear mixed effects model 1 (above) for juvenile survival probabilities (*P-adjusted* value = two-sided Tukey post-hoc tests).

Treatment x Treatment comparison		Estimate	Std. Error	z value	Pr(>|z|)
32.C1 -	26.C1	-0.229299	0.066668	-3.439	0.0134 *
26.D1 -	26.C1	-0.057231	0.066493	-0.861	0.9894
32.D1 -	26.C1	-0.117873	0.066496	-1.773	0.6385
26.SED -	26.C1	-0.14995	0.066695	-2.248	0.3232
32.SED -	26.C1	-0.146693	0.066744	-2.198	0.3529
26.SS -	26.C1	-0.079034	0.066558	-1.187	0.9358
32.SS -	26.C1	-0.139358	0.06667	-2.09	0.421
26.D1 -	32.C1	0.172069	0.06676	2.577	0.1644
32.D1 -	32.C1	0.111426	0.066763	1.669	0.7074
26.SED -	32.C1	0.079349	0.066963	1.185	0.9364
32.SED -	32.C1	0.082606	0.067006	1.233	0.9223
26.SS -	32.C1	0.150266	0.066816	2.249	0.3223
32.SS -	32.C1	0.089942	0.06693	1.344	0.8822
32.D1 -	26.D1	-0.060643	0.066583	-0.911	0.9851
26.SED -	26.D1	-0.092719	0.066781	-1.388	0.8629
32.SED -	26.D1	-0.089463	0.066832	-1.339	0.8842
26.SS -	26.D1	-0.021803	0.066638	-0.327	1
32.SS -	26.D1	-0.082127	0.066751	-1.23	0.9231
26.SED -	32.D1	-0.032077	0.066789	-0.48	0.9997
32.SED -	32.D1	-0.02882	0.066836	-0.431	0.9999
26.SS -	32.D1	0.03884	0.066648	0.583	0.9991
32.SS -	32.D1	-0.021484	0.066754	-0.322	1
32.SED -	26.SED	0.003257	0.067035	0.049	1
26.SS -	26.SED	0.070916	0.066843	1.061	0.9647
32.SS -	26.SED	0.010593	0.066966	0.158	1
26.SS -	32.SED	0.06766	0.066888	1.012	0.9729
32.SS -	32.SED	0.007336	0.066998	0.109	1
32.SS -	26.SS	-0.060324	0.066814	-0.903	0.9859

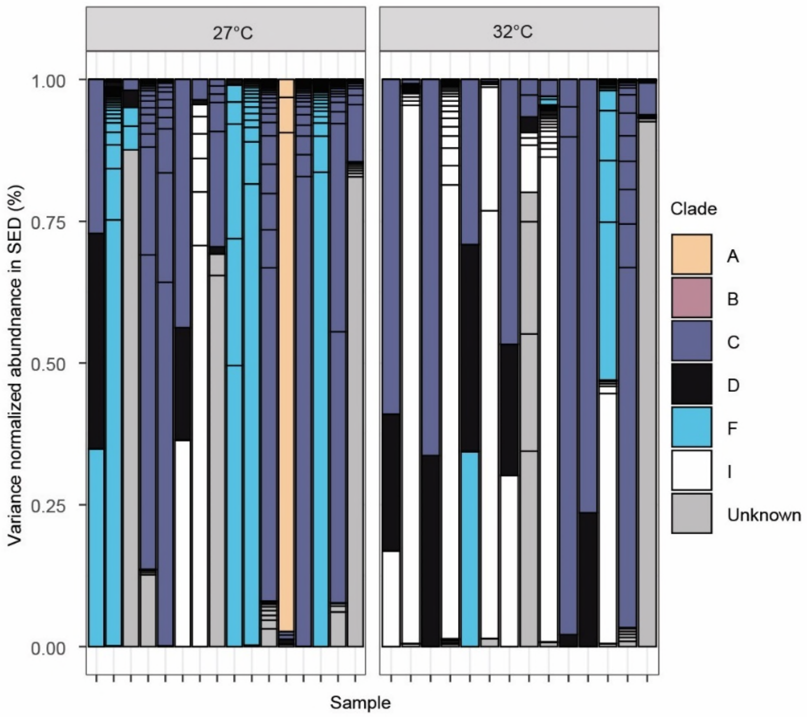

Supplementary Fig. 1. Symbiodiniaceae community composition in purebred and hybrid juvenile corals exposed to sediment (SED) communities and subjected to 27°C and heat treatments. Amplicon sequencing confirmed symbiont identities in single juveniles revealed a high diversity of symbionts in juveniles exposed to communities in the sediments collected from hot, inshore Curd reef. Colours correspond to Symbiodiniaceae taxonomic groupings ("Clades"), which broadly correspond to Genera.

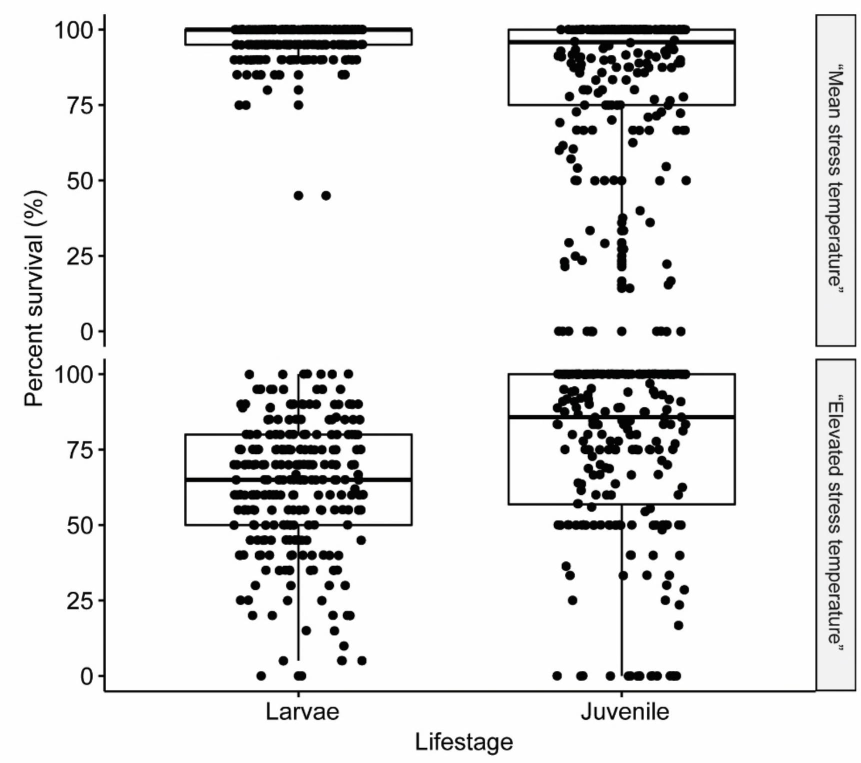

Supplementary Fig. 2. Boxplots comparing percent larval survival to percent (%) juvenile survival at the final timepoint in which the median value is calculated over all crosses at 27°C and the elevated stress temperature treatments. Derived statistics presented as box plots are defined as independent observations of n= independent larval survival based on the number of remaining individuals per replicate well at the final sampling time point. Boxplots include the median values (center lines), upper and lower quartiles (box limits), 1.5x interquartile range (whiskers), and outliers (points).

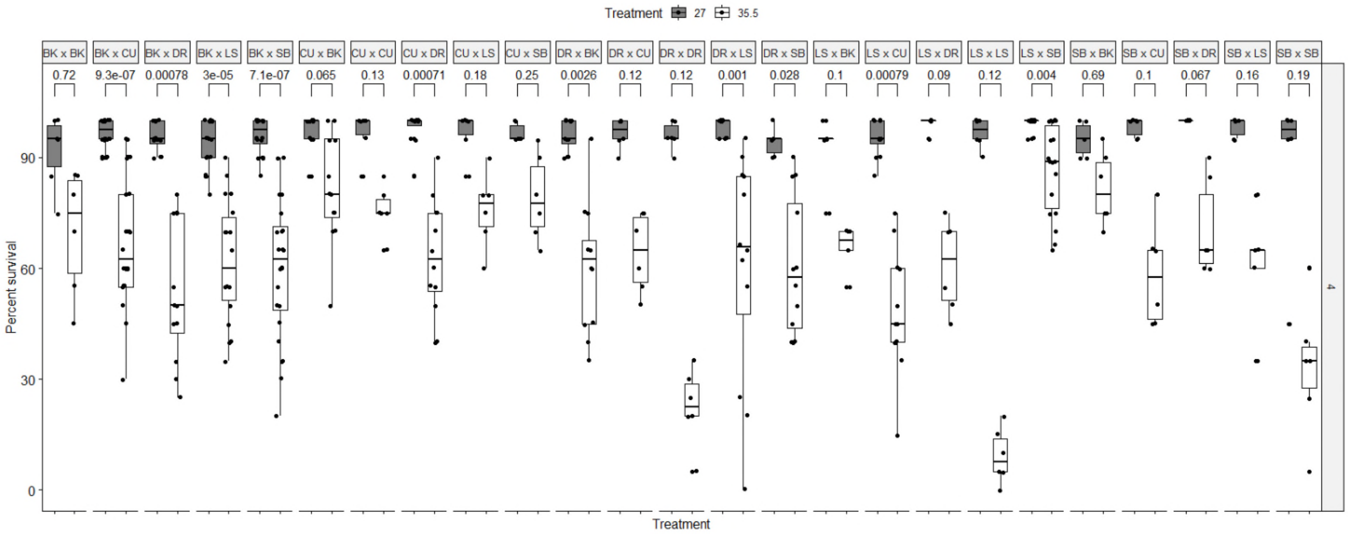

Supplementary Fig. 3. Boxplots comparing percent survival of larvae from different purebred and hybrid crosses at the final timepoint (56 hours) between each cross at 27 (grey) and 35°C (white). P.adj-values above the boxplots correspond to non-parametric, two-sided Wilcox test results. Statistical differences between treatments are shown (Bonferroni post-hoc test for multiple pairwise comparisons). Derived statistics presented as box plots are defined as independent observations of n= independent larval survival based on the number of remaining individuals per replicate well at the final sampling time point. Boxplots include the median values (center lines), upper and lower quartiles (box limits), 1.5x interquartile range (whiskers), and outliers (points).

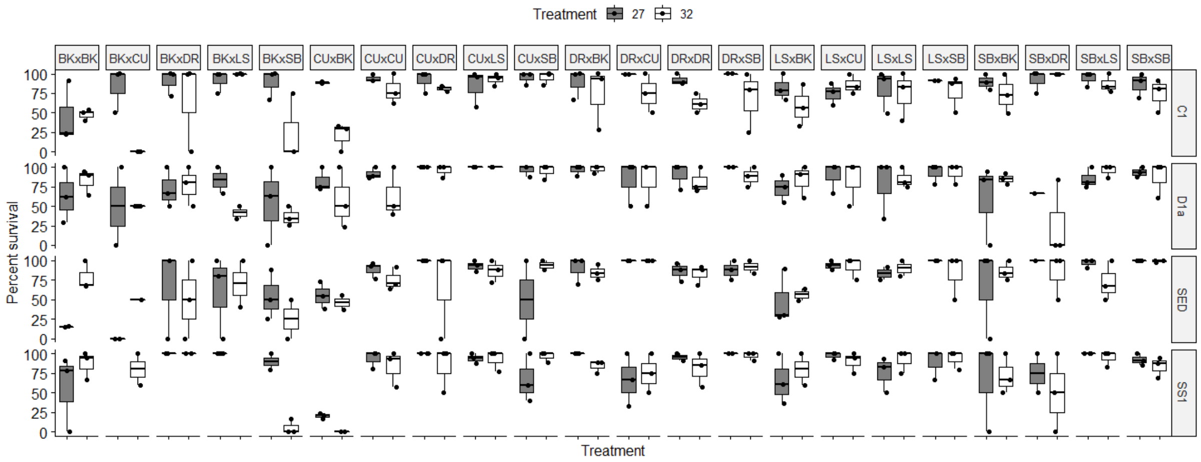

Supplementary Fig. 4. Boxplots comparing percent juvenile survival at the final timepoint (58 days) between each cross at 27 (grey) and 32°C (white). Values above the boxplots correspond to values derived from Bonferroni p-adjusted values for multiple comparisons using non-parametric two-sided Wilcox tests. Note, no single or clumped juveniles settled for the SS treatment at 27°C for BKxCU or 32°C for BKxLS, labelled as "/" . Crosses labelled as "NA" are due to the Wilcox test not computing the test statistic for complete ties, i.e. all three replicates per treatment were the same at survival = 100%. Both temperature treatments at 27 and 32°C resulted in 100% survival for CUxLS – D1a, DRxCU – SED, BKxDR – SS1. There were no statistical differences between any cross treatments after Bonferroni post-hoc tests for multiple pairwise comparisons were performed. Boxplots include the median values (center lines), upper and lower quartiles (box limits), 1.5x interquartile range (whiskers), and outliers (points).

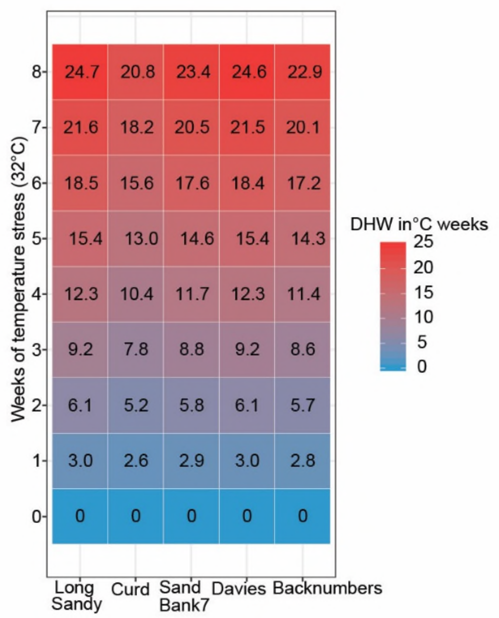

Supplementary Fig. 5. Estimated level of experimental thermal stress experienced in the 32°C treatment for juveniles sourced for the five reefs used in this study. Heatmap colours represent Degree Heating Weeks (DHW).

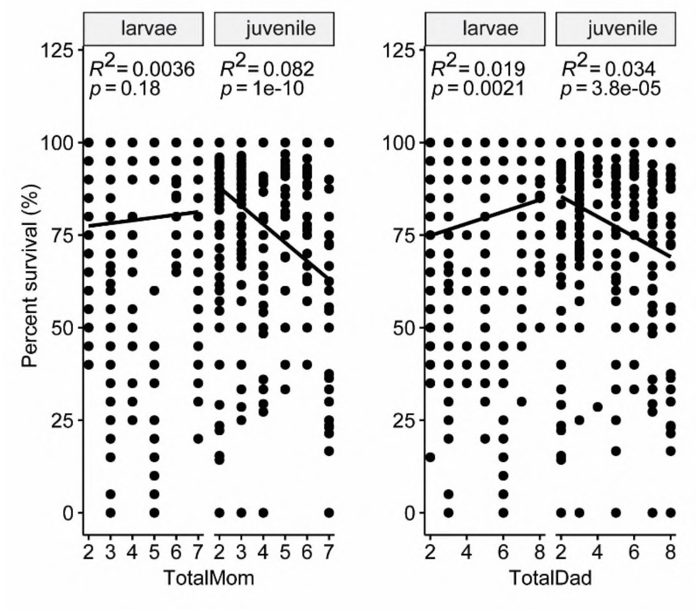

Supplementary Fig. 6. Correlation between the number of unique maternal or paternal colonies used in each cross compared to percent (%) survival in larvae and juveniles. R^2 and p-values correspond to Pearson correlation coefficients.

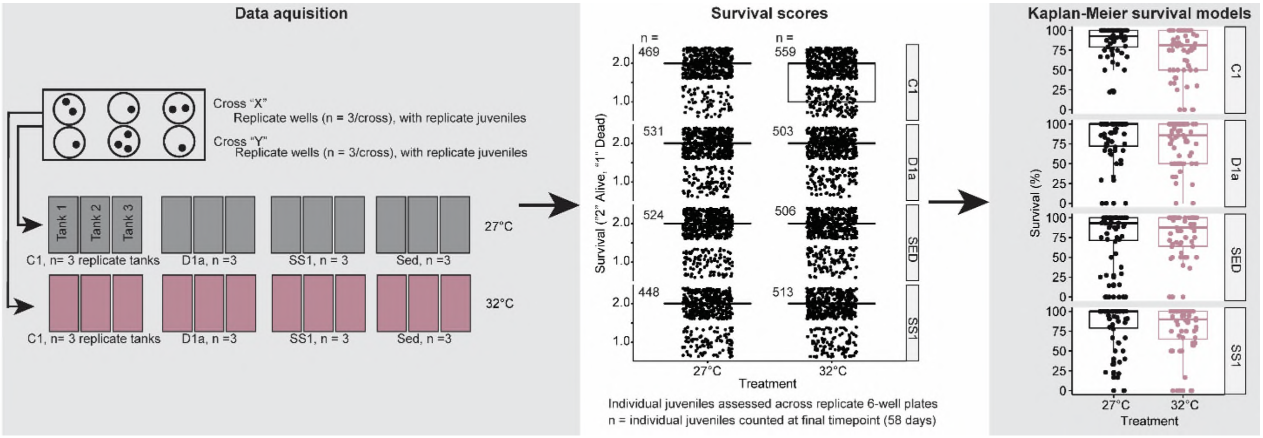

Supplementary Fig. 7. Experimental design summary for the processing of juvenile percent (%) survival data, shaded by three steps (data acquisition, survival scores from photographs, and survival models). Colours correspond to temperature treatments at 27 (dark grey) and 32°C (pink).

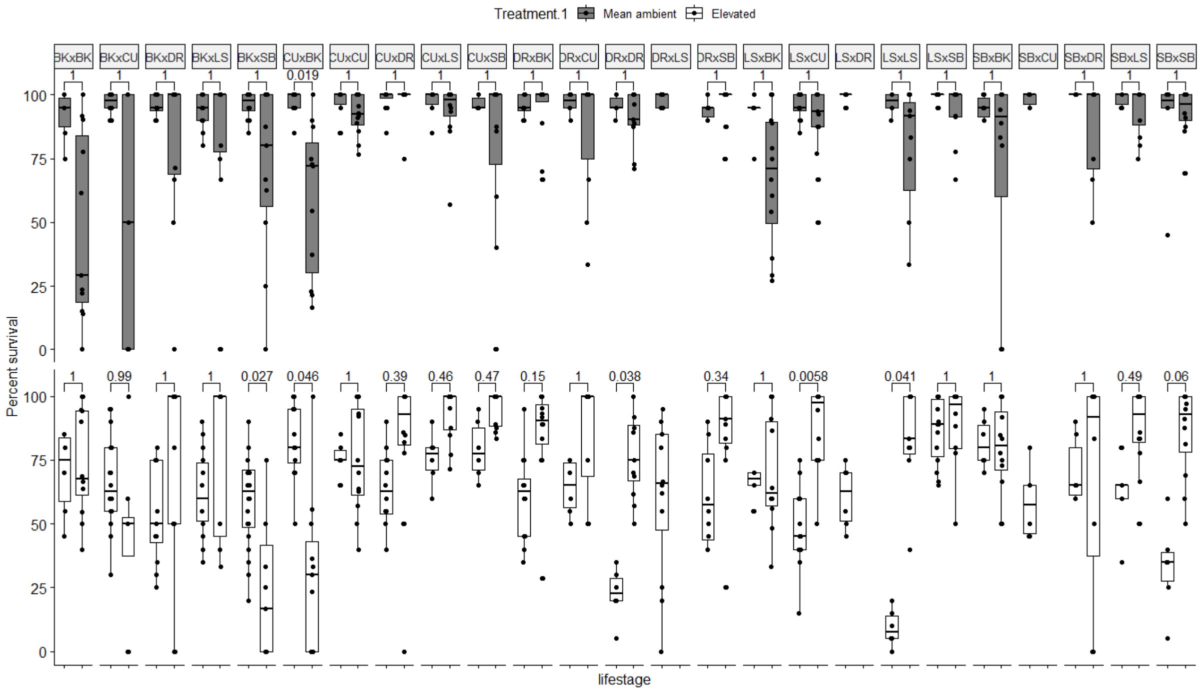

Supplementary Fig. 8. Boxplots comparing percent larval survival to percent juvenile survival at the final timepoint per cross at 27 (grey) and 32°C (white). Values above the boxplots correspond to Bonferroni p-adjusted values for multiple comparisons derived from the non-parametric two-sided Wilcox test. Some crosses were not available at the juvenile stage, either because those crosses either did not settle or died or were not used due to space limitations during the juvenile settlement experiment (these are not labelled, including DRxLS, LSxDR, SBxCU). Derived statistics presented as box plots are defined as independent observations of n= independent larval or juvenile survival based on the number of remaining individuals per replicate well at the final sampling time point. Boxplots include the median values (center lines), upper and lower quartiles (box limits), 1.5x interquartile range (whiskers), and outliers (points).

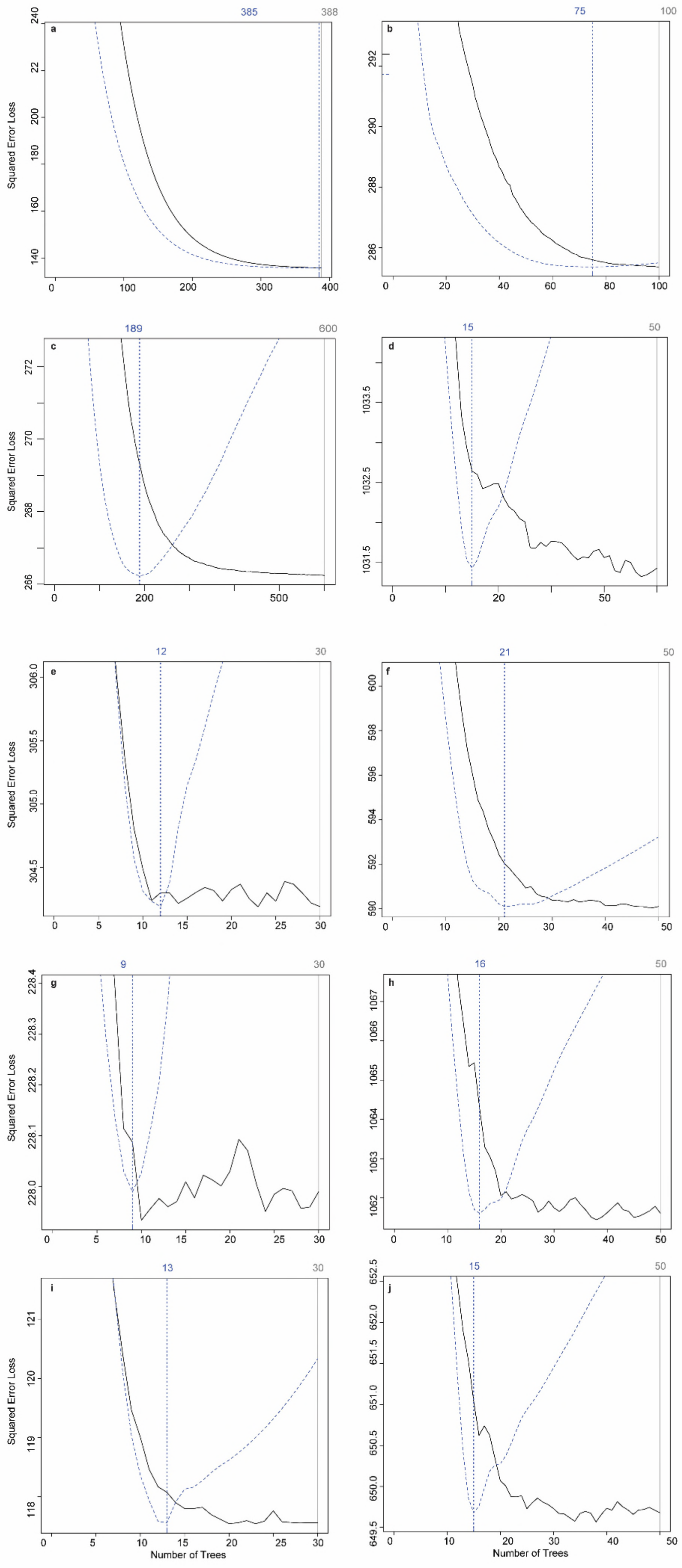
a
385
388
Squared Error Loss
b
75
100
c
189
600
d
15
50
e
12
30
f
21
50
g
9
30
h
16
50
i
13
30
j
15
50
Number of Trees

Supplementary Fig. 9. Diagnostic plots of Boosted Regression Tree hyperparameters. These are included for each of the experimental groups: aposymbiotic purebred and hybrid larvae (**a, b**), juveniles exposed to C1 (**c, d**), D1a (**e, f**), SS1 (**g, h**), and the sediment treatment (**i, j**).

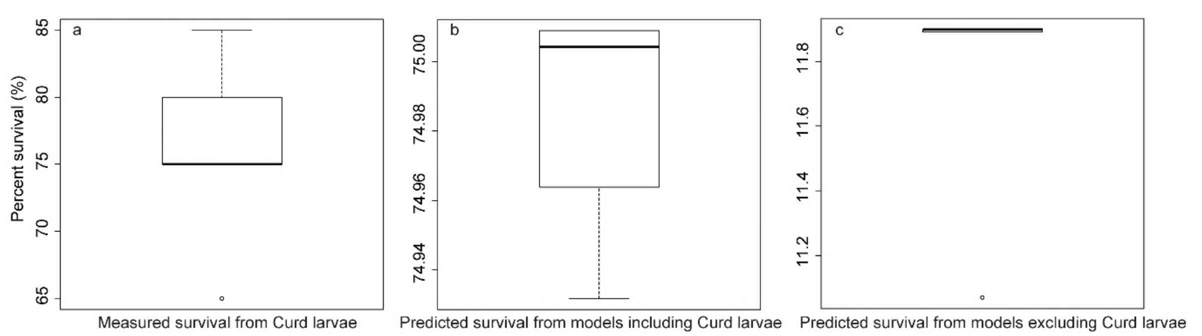

Supplementary Fig. 10. Simulated ability of the models to predict with or without whole reefs removed from dataset. Compared to the raw data of survival measured in Curd purebred larvae (**a**), the models were able to predict the percent (%) survival of Curd reef larvae well when all reefs are included (**b**). However, if whole reefs are removed (**c**, Curd reef in this example), survival is underestimated. Boxplots include the median values (center lines), upper and lower quartiles (box limits), 1.5x interquartile range (whiskers), and outliers (points).

References used in the Supplementary Material

1. Randall, C. J. *et al.* Sexual production of corals for reef restoration in the Anthropocene. *Marine Ecology Progress Series* (2020). doi:10.3354/MEPS13206
2. Hughes, T. P. *et al.* Global warming and recurrent mass bleaching of corals. *Nature* **543**, 373–377 (2017).
3. Quigley, K. M., Willis, B. L. & Bay, L. K. Heritability of the *Symbiodinium* community in vertically-and horizontally-transmitting broadcast spawning corals. *Sci. Rep.* (2017). doi:10.1038/s41598-017-08179-4
4. Edgar, R. C. UPARSE: highly accurate OTU sequences from microbial amplicon reads. *Nat. Methods* **10**, 996–998 (2013).
5. Quigley, K. M. *et al.* Deep-sequencing method for quantifying background abundances of Symbiodinium types: Exploring the rare *Symbiodinium* biosphere in reef-building corals. *PLoS One* **9**, (2014).
6. Callahan, B. J. *et al.* DADA2: high-resolution sample inference from Illumina amplicon data. *Nat. Methods* **13**, 581–583 (2016).
7. Quigley, K. M., Willis, B. L. & Kenkel, C. D. Transgenerational inheritance of shuffled symbiont communities in the coral *Montipora digitata*. *Sci. Rep.* **9**, (2019).
8. Quigley, K. M. *et al.* Variability in fitness trade-offs amongst coral juveniles with mixed genetic backgrounds held in the wild. *Front. Mar. Sci.* **8**, 161 (2021).

9. Saha, Korak; Zhao, Xuepeng; Zhang, Huai-min; Casey, Kenneth S.; Zhang, Dexin; Zhang, Yongsheng; Baker-Yeboah, Sheekela; Relph, John M.; Krishnan, Ajay; Ryan, T. The Coral Reef Temperature Anomaly Database (CoRTAD) Version 6 - Global, 4 km Sea Surface Temperature and Related Thermal Stress Metrics for 1982 to 2019. *NOAA Natl. Centers Environ. Inf.* (2018).

10. Steven, A. D. L. *et al.* eReefs: An operational information system for managing the Great Barrier Reef. *J. Oper. Oceanogr.* **12**, S12–S28 (2019).
