# Supplementary material for: Predictive models for the selection of thermally tolerant corals based on offspring survival
Source: Nat Commun. 2022 Mar 29;13:1543. doi: 10.1038/s41467-022-28956-8 (PMC8964693; doi:10.1038/s41467-022-28956-8)
Supplement: Supplementary file 3 — Description of Additional Supplementary Information [file 41467_2022_28956_MOESM3_ESM.docx]

Description of Additional Supplementary Files

**Title: Supplementary Data 1.**

**Description:** Summary of the exact sample sizes of larval survival replicates in each of the crosses from 0 to 56 hours at 27°C and 35.5°C treatments as presented in Fig. 2a. The exact samples size (*n*) is listed in column 6 for each experimental group. Each represents a discrete sample measurement. The unit of measure is the number of individual replicate wells containing larvae.

**Title: Supplementary Data 2.**

**Description:** Summary of the exact sample sizes of juvenile survival in each of the crosses at the start and end of the experiment (after 58 days) at 27 and 32°C treatments as presented in Fig. 2a. The exact samples size (*n*) is listed in column 8 for each experimental group. Each represents a discrete sample measurement. The unit of measure is the number of individual replicate juveniles per replicate well, per replicate plate, per replicate tank for each temperature and symbiont treatment.
