# Supplementary material for: Predictive models for the selection of thermally tolerant corals based on offspring survival
Source: Nat Commun. 2022 Mar 29;13:1543. doi: 10.1038/s41467-022-28956-8 (PMC8964693; doi:10.1038/s41467-022-28956-8)
Supplement: Supplementary file 6 — Reporting Summary [file 41467_2022_28956_MOESM6_ESM.pdf]

nature portfolio

Corresponding author(s): Kate Quigley

Last updated by author(s): Feb 11, 2022

Reporting Summary

Nature Portfolio wishes to improve the reproducibility of the work that we publish. This form provides structure for consistency and transparency in reporting. For further information on Nature Portfolio policies, see our Editorial Policies and the Editorial Policy Checklist.

Statistics

For all statistical analyses, confirm that the following items are present in the figure legend, table legend, main text, or Methods section.

n/a	Confirmed	
☐	☒	The exact sample size (*n*) for each experimental group/condition, given as a discrete number and unit of measurement
☐	☒	A statement on whether measurements were taken from distinct samples or whether the same sample was measured repeatedly
☐	☒	The statistical test(s) used AND whether they are one- or two-sided<br>*Only common tests should be described solely by name; describe more complex techniques in the Methods section.*
☐	☒	A description of all covariates tested
☐	☒	A description of any assumptions or corrections, such as tests of normality and adjustment for multiple comparisons
☐	☒	A full description of the statistical parameters including central tendency (e.g. means) or other basic estimates (e.g. regression coefficient) AND variation (e.g. standard deviation) or associated estimates of uncertainty (e.g. confidence intervals)
☐	☒	For null hypothesis testing, the test statistic (e.g. *F*, *t*, *r*) with confidence intervals, effect sizes, degrees of freedom and *P* value noted<br>*Give P values as exact values whenever suitable.*
☒	☐	For Bayesian analysis, information on the choice of priors and Markov chain Monte Carlo settings
☐	☒	For hierarchical and complex designs, identification of the appropriate level for tests and full reporting of outcomes
☐	☒	Estimates of effect sizes (e.g. Cohen's *d*, Pearson's *r*), indicating how they were calculated

Our web collection on statistics for biologists contains articles on many of the points above.

Software and code

Policy information about availability of computer code

Data collection

Datasets included and provided on Github include:

AGF_yr2_Juvs_cur.singles_aliveatT1_onlySurv_Github.csv
AGF18.Sites_Github.csv
ARCGIS_KQuigley_Satdata_AGF18sites_CSVlatlong.csv
GBRONLYSSTAExport_Output_csv.csv
ITS2_AGF18_Ariftax_Edit.csv
Juveniles_AGF18_Phyloseq_Metadata.csv
larv_juv_comparision_combo.csv
larv6.csv
larvaldataAGF2018_Github.csv
RelativeImportance_summaryAGF18.csv
juv6.6_subset.zip

Sequencing data are available at NCBI SRA at PRJNA720058 and analysis scripts for ITS-2 data are found at are located at: https://github.com/LaserKate/AGF18_MachineLearning.git. This "Data availability" statement is included in the manuscript.

Data analysis

All analysis information and code can be found on: https://github.com/LaserKate/AGF18_MachineLearning.git.
The following software and packages used are listed below:
R (v.3.6.0)
ereefs- 2.6.10
ncdf4- 1.17

rlang - 0.4.10
caret -6.0-86
gbm -2.1.5
rsample- 0.0.6
dplyr- 0.8.0.1
plotmo-3.5.7
pdp-0.7.0
ggpubr-0.1.8
patchwork-1.0.0
mapping-0.1
maptools- 0.9-9
scales-1.1.1
dismo-1.1-4
mapping-0.1
sp-1.3-1
tidyr-1.0.3
ggrepel- 0.8.2
ggpubr-0.3.0
survival-2.44-1.1
broom-0.5.6
stringr-1.4.0
multcomp-1.4-10

For manuscripts utilizing custom algorithms or software that are central to the research but not yet described in published literature, software must be made available to editors and reviewers. We strongly encourage code deposition in a community repository (e.g. GitHub). See the Nature Portfolio guidelines for submitting code & software for further information.

Data

Policy information about availability of data

All manuscripts must include a data availability statement. This statement should provide the following information, where applicable:

- Accession codes, unique identifiers, or web links for publicly available datasets
- A description of any restrictions on data availability
- For clinical datasets or third party data, please ensure that the statement adheres to our policy

Data are available from K.Quigley's Github (https://github.com/LaserKate/AGF18_MachineLearning.git). Amplicon sequencing data of the Symbiodiniaceae ITS2 region data is available on the Sequence Read Archive (BioProject PRJNA720058).

Field-specific reporting

Please select the one below that is the best fit for your research. If you are not sure, read the appropriate sections before making your selection.

☐ Life sciences ☐ Behavioural & social sciences ☒ Ecological, evolutionary & environmental sciences

For a reference copy of the document with all sections, see nature.com/documents/nr-reporting-summary-flat.pdf

Life sciences study design

All studies must disclose on these points even when the disclosure is negative.

Sample size	*Describe how sample size was determined, detailing any statistical methods used to predetermine sample size OR if no sample-size calculation was performed, describe how sample sizes were chosen and provide a rationale for why these sample sizes are sufficient.*
Data exclusions	*Describe any data exclusions. If no data were excluded from the analyses, state so OR if data were excluded, describe the exclusions and the rationale behind them, indicating whether exclusion criteria were pre-established.*
Replication	*Describe the measures taken to verify the reproducibility of the experimental findings. If all attempts at replication were successful, confirm this OR if there are any findings that were not replicated or cannot be reproduced, note this and describe why.*
Randomization	*Describe how samples/organisms/participants were allocated into experimental groups. If allocation was not random, describe how covariates were controlled OR if this is not relevant to your study, explain why.*
Blinding	*Describe whether the investigators were blinded to group allocation during data collection and/or analysis. If blinding was not possible, describe why OR explain why blinding was not relevant to your study.*

Behavioural & social sciences study design

All studies must disclose on these points even when the disclosure is negative.

Study description	*Briefly describe the study type including whether data are quantitative, qualitative, or mixed-methods (e.g. qualitative cross-sectional, quantitative experimental, mixed-methods case study).*
Research sample	*State the research sample (e.g. Harvard university undergraduates, villagers in rural India) and provide relevant demographic information (e.g. age, sex) and indicate whether the sample is representative. Provide a rationale for the study sample chosen. For studies involving existing datasets, please describe the dataset and source.*
Sampling strategy	*Describe the sampling procedure (e.g. random, snowball, stratified, convenience). Describe the statistical methods that were used to predetermine sample size OR if no sample-size calculation was performed, describe how sample sizes were chosen and provide a rationale for why these sample sizes are sufficient. For qualitative data, please indicate whether data saturation was considered, and what criteria were used to decide that no further sampling was needed.*
Data collection	*Provide details about the data collection procedure, including the instruments or devices used to record the data (e.g. pen and paper, computer, eye tracker, video or audio equipment) whether anyone was present besides the participant(s) and the researcher, and whether the researcher was blind to experimental condition and/or the study hypothesis during data collection.*
Timing	*Indicate the start and stop dates of data collection. If there is a gap between collection periods, state the dates for each sample cohort.*
Data exclusions	*If no data were excluded from the analyses, state so OR if data were excluded, provide the exact number of exclusions and the rationale behind them, indicating whether exclusion criteria were pre-established.*
Non-participation	*State how many participants dropped out/declined participation and the reason(s) given OR provide response rate OR state that no participants dropped out/declined participation.*
Randomization	*If participants were not allocated into experimental groups, state so OR describe how participants were allocated to groups, and if allocation was not random, describe how covariates were controlled.*

Ecological, evolutionary & environmental sciences study design

All studies must disclose on these points even when the disclosure is negative.

Study description	Derived statistics presented as box plots are defined as independent observations of n= independent larval or juvenile survival based on the number of remaining individuals per replicate well at the final sampling time point.<br><br>Experimental table of detailed replicates for larval treatments are found in Supplementary Table 4. Total n = 2220 of larval replicate counts.<br>Experimental design for juvenile data is found in Supplementary Figure 7 and Supplementary Table 5. Total n = 12,171 observations of individual juveniles.<br><br>Wild corals were collected throughout the Great Barrier Reef and reproductively crossed at a land based facility. Larvae were reared and subjected to a heat test. Larvae were then induced to settle, then exposed to symbionts and then exposed to heat stress. Both larvae and juveniles were sampled for survival. The experimental design was hierarchical.<br>Larval replicates were measured for each of the crosses from 0 to 56 hours at 27°C and 35.5°C treatments. Individual larvae were counted within replicate net-wells, in replicate, plates within each temperature treatment. Each larval survival measurement represents a discrete sample measurement. The unit of measure is the number of individual replicate wells containing larvae.<br>Juvenile replicates were measured in each of the crosses at the start and end of the experiment (after 58 days) at 27 and 32°C. Each measurement represents individual juvenile survival. Each represents a discrete sample measurement. The unit of measure is the number of individual replicate juveniles per replicate well, per replicate plate, per replicate tank for each temperature and symbiont treatment.
Research sample	A research sample involved individual genotypes of corals from different life-stages. For example, a survival measurement of a single larvae or a single juvenile coral. Phenotypes were measured directly on the individual basis. Individual samples were collected as it provided greater power to the experimental design compared to bulk measurements. The species used was Acropora tenuis. Adults were collected from across the northern and central Great Barrier Reef (coordinates provided), of at least 3 to 5 years of age, wild-caught. Both larvae and juveniles were reproductive crossed (manipulated) in a quantitative genetic cross design. This species was chosen given its amenability to coral reproductive biology and settlement cues, necessary for the experiment.
Sampling strategy	Counting and sampling surviving coral larvae and juveniles after their experimental exposure to heat stress was conducted using established methods in the field.<br>Sample sizes were chosen for larvae to maximize the number of larvae per well for DNA and RNA extraction (n = 30) but still allowed for accurate counting (optimum is 10).<br>Sample size were chosen for juveniles to optimize the number of juveniles that could be induced to metamorphose whilst maximizing the probability of at least n = 5 individuals surviving by the end of the experiment.

Data collection	Physiological data on survival was collected at the larval and juvenile stage.<br>Larval counts were conducted by having one person with a light standing over the wells and counting the number of individuals swimming, whilst a second person recorded the data. Juveniles were photographed with a Nikon D810 with a Nikon AF-S 60 mm f/2.8 G Micro ED Lens with four Ikelite DS160 Strobes. Photographs were analyzed in ImageJ.
Timing and spatial scale	Larval survival was counted from 0 to 56 hours at 27°C and 35.5°C.<br>Juvenile survival was counted at 0 and after 58 days at 27 and 32°C treatments.<br>The spatial scale for which these colonies were collected were from latitudes -12.50029 (degS) to -18.821744 (degS). See manuscript for map of locations. Experiments were carried out from Nov 2018 until March 2019.<br>Counts and sampling was performed when the average survival across all crosses in the heat treatment reached approximately 50%.
Data exclusions	No data were excluded. All data are found via Github.
Reproducibility	Replicate biological crosses were made within the experiment. These are biological replicates that are included as replicates within the statistical design. This experiment has not been repeated to verify reproducibility.
Randomization	Randomization of individual larvae and juveniles was carried across across all replicate wells, plates, tanks, and temperatures.
Blinding	Given the randomization of familial crosses within the larval and juvenile experiments, it was impossible to know which family was being counted (therefore blind to cross identity). However, we could not blind to tank temperature as when you are counting you can feel the difference in temperature between ambient and warm treatments). Symbiont identity was also not blinded for the juvenile experiment as the phenotypes of juveniles between the four treatments was very different.

Did the study involve field work? ☒ Yes ☐ No

Field work, collection and transport

Field conditions	The relevant temperature profiles per study site (location of coral collections) are described in detail in Figure 1.
Location	Great Barrier Reef (Australia). The field parameters of each site are described extensively in Figure 1.<br>These included, in the north, Curd (CU: −12.5850∘S, 143.5115∘E), Sand Bank 7 (SB: −13.4362∘S, 143.9714∘E), Long Sandy (LS: −12.5003∘S, 143.7848∘E), Davies (DR: −18.8217∘S, 147.6495∘E), and Backnumbers (BK: −18.5075∘S, 147.1464∘E) reefs in the central region. Water depth of collections ~4m.
Access & import/export	All field work and collection and care of laboratory animals were conducted under institutional guidelines and permit number G18/41667.1.
Disturbance	Colonies were collected from the reef without disturbing the surrounding benthos as per permit conditions.

Reporting for specific materials, systems and methods

We require information from authors about some types of materials, experimental systems and methods used in many studies. Here, indicate whether each material, system or method listed is relevant to your study. If you are not sure if a list item applies to your research, read the appropriate section before selecting a response.

Materials & experimental systems

n/a	Involved in the study	
☒	☐	Antibodies
☒	☐	Eukaryotic cell lines
☒	☐	Palaeontology and archaeology
☐	☒	Animals and other organisms
☒	☐	Human research participants
☒	☐	Clinical data
☒	☐	Dual use research of concern

Methods

n/a	Involved in the study	
☒	☐	ChIP-seq
☒	☐	Flow cytometry
☒	☐	MRI-based neuroimaging

Antibodies

Antibodies used	*Describe all antibodies used in the study; as applicable, provide supplier name, catalog number, clone name, and lot number.*
Validation	*Describe the validation of each primary antibody for the species and application, noting any validation statements on the manufacturer's website, relevant citations, antibody profiles in online databases, or data provided in the manuscript.*

Eukaryotic cell lines

Policy information about cell lines

Cell line source(s)	*State the source of each cell line used.*
Authentication	*Describe the authentication procedures for each cell line used OR declare that none of the cell lines used were authenticated.*
Mycoplasma contamination	*Confirm that all cell lines tested negative for mycoplasma contamination OR describe the results of the testing for mycoplasma contamination OR declare that the cell lines were not tested for mycoplasma contamination.*
Commonly misidentified lines (See ICLAC register)	*Name any commonly misidentified cell lines used in the study and provide a rationale for their use.*

Palaeontology and Archaeology

Specimen provenance	*Provide provenance information for specimens and describe permits that were obtained for the work (including the name of the issuing authority, the date of issue, and any identifying information). Permits should encompass collection and, where applicable, export.*
Specimen deposition	*Indicate where the specimens have been deposited to permit free access by other researchers.*
Dating methods	*If new dates are provided, describe how they were obtained (e.g. collection, storage, sample pretreatment and measurement), where they were obtained (i.e. lab name), the calibration program and the protocol for quality assurance OR state that no new dates are provided.*

☐ Tick this box to confirm that the raw and calibrated dates are available in the paper or in Supplementary Information.

Ethics oversight	*Identify the organization(s) that approved or provided guidance on the study protocol, OR state that no ethical approval or guidance was required and explain why not.*

Note that full information on the approval of the study protocol must also be provided in the manuscript.

Animals and other organisms

Policy information about studies involving animals; ARRIVE guidelines recommended for reporting animal research

Laboratory animals	Laboratory animals were produced by reproductively crossing field-collected, wild animals. All animals were from Acropora tenuis.
Wild animals	Wild caught reproductive adults of Acropora tenuis were collected from the field. Each likely around 3 to 5 years of age. Individual corals were collected using chisel and hammer from the reef by divers on SCUBA. Animals were transported via plane or boat to the aquarium facility and house in separate tanks set to the daily temperature variation cycle of their reef of origin. Adults were killed during a mass-flooding, extreme-weather event at the facility after these experiments.
Field-collected samples	Wild caught, field-collected reproductive adults of Acropora tenuis were collected from the field. Each likely around 3 to 5 years of age. Individual corals were collected using chisel and hammer from the reef by divers on SCUBA. Animals were transported via plane or boat to the aquarium facility and house in separate tanks set to the daily temperature variation cycle of their reef of origin. Adults were killed during a mass-flooding, extreme-weather event at the facility after these experiments. Corals were collected between 9 am and 4 pm from the field. Temperature profiles for each of these sites is described in depth in Figure 1.
Ethics oversight	Corals are not subject to Ethics oversight within Australia. All field work and collection and care of laboratory animals were conducted under institutional guidelines and permit number G18/41667.1.

Note that full information on the approval of the study protocol must also be provided in the manuscript.

Human research participants

Policy information about studies involving human research participants

Population characteristics	*Describe the covariate-relevant population characteristics of the human research participants (e.g. age, gender, genotypic information, past and current diagnosis and treatment categories). If you filled out the behavioural & social sciences study design questions and have nothing to add here, write "See above."*
Recruitment	*Describe how participants were recruited. Outline any potential self-selection bias or other biases that may be present and how these are likely to impact results.*
Ethics oversight	*Identify the organization(s) that approved the study protocol.*

Note that full information on the approval of the study protocol must also be provided in the manuscript.

Clinical data

Policy information about clinical studies

All manuscripts should comply with the ICMJE guidelines for publication of clinical research and a completed CONSORT checklist must be included with all submissions.

Clinical trial registration	*Provide the trial registration number from ClinicalTrials.gov or an equivalent agency.*
Study protocol	*Note where the full trial protocol can be accessed OR if not available, explain why.*
Data collection	*Describe the settings and locales of data collection, noting the time periods of recruitment and data collection.*
Outcomes	*Describe how you pre-defined primary and secondary outcome measures and how you assessed these measures.*

Dual use research of concern

Policy information about dual use research of concern

Hazards

Could the accidental, deliberate or reckless misuse of agents or technologies generated in the work, or the application of information presented in the manuscript, pose a threat to:

No	Yes	
☐	☐	Public health
☐	☐	National security
☐	☐	Crops and/or livestock
☐	☐	Ecosystems
☐	☐	Any other significant area

Experiments of concern

Does the work involve any of these experiments of concern:

No	Yes	
☐	☐	Demonstrate how to render a vaccine ineffective
☐	☐	Confer resistance to therapeutically useful antibiotics or antiviral agents
☐	☐	Enhance the virulence of a pathogen or render a nonpathogen virulent
☐	☐	Increase transmissibility of a pathogen
☐	☐	Alter the host range of a pathogen
☐	☐	Enable evasion of diagnostic/detection modalities
☐	☐	Enable the weaponization of a biological agent or toxin
☐	☐	Any other potentially harmful combination of experiments and agents

ChIP-seq

Data deposition

☐ Confirm that both raw and final processed data have been deposited in a public database such as GEO.

☐ Confirm that you have deposited or provided access to graph files (e.g. BED files) for the called peaks.

Data access links<br>*May remain private before publication.*	*For "Initial submission" or "Revised version" documents, provide reviewer access links. For your "Final submission" document, provide a link to the deposited data.*
Files in database submission	*Provide a list of all files available in the database submission.*
Genome browser session<br>(e.g. UCSC)	*Provide a link to an anonymized genome browser session for "Initial submission" and "Revised version" documents only, to enable peer review. Write "no longer applicable" for "Final submission" documents.*

Methodology

Replicates	*Describe the experimental replicates, specifying number, type and replicate agreement.*
Sequencing depth	*Describe the sequencing depth for each experiment, providing the total number of reads, uniquely mapped reads, length of reads and whether they were paired- or single-end.*

Antibodies	*Describe the antibodies used for the ChIP-seq experiments; as applicable, provide supplier name, catalog number, clone name, and lot number.*
Peak calling parameters	*Specify the command line program and parameters used for read mapping and peak calling, including the ChIP, control and index files used.*
Data quality	*Describe the methods used to ensure data quality in full detail, including how many peaks are at FDR 5% and above 5-fold enrichment.*
Software	*Describe the software used to collect and analyze the ChIP-seq data. For custom code that has been deposited into a community repository, provide accession details.*

Flow Cytometry

Plots

Confirm that:

- [ ] The axis labels state the marker and fluorochrome used (e.g. CD4-FITC).
- [ ] The axis scales are clearly visible. Include numbers along axes only for bottom left plot of group (a 'group' is an analysis of identical markers).
- [ ] All plots are contour plots with outliers or pseudocolor plots.
- [ ] A numerical value for number of cells or percentage (with statistics) is provided.

Methodology

Sample preparation	*Describe the sample preparation, detailing the biological source of the cells and any tissue processing steps used.*
Instrument	*Identify the instrument used for data collection, specifying make and model number.*
Software	*Describe the software used to collect and analyze the flow cytometry data. For custom code that has been deposited into a community repository, provide accession details.*
Cell population abundance	*Describe the abundance of the relevant cell populations within post-sort fractions, providing details on the purity of the samples and how it was determined.*
Gating strategy	*Describe the gating strategy used for all relevant experiments, specifying the preliminary FSC/SSC gates of the starting cell population, indicating where boundaries between "positive" and "negative" staining cell populations are defined.*

- [ ] Tick this box to confirm that a figure exemplifying the gating strategy is provided in the Supplementary Information.

Magnetic resonance imaging

Experimental design

Design type	*Indicate task or resting state; event-related or block design.*
Design specifications	*Specify the number of blocks, trials or experimental units per session and/or subject, and specify the length of each trial or block (if trials are blocked) and interval between trials.*
Behavioral performance measures	*State number and/or type of variables recorded (e.g. correct button press, response time) and what statistics were used to establish that the subjects were performing the task as expected (e.g. mean, range, and/or standard deviation across subjects).*

Acquisition

Imaging type(s)	*Specify: functional, structural, diffusion, perfusion.*
Field strength	*Specify in Tesla*
Sequence & imaging parameters	*Specify the pulse sequence type (gradient echo, spin echo, etc.), imaging type (EPI, spiral, etc.), field of view, matrix size, slice thickness, orientation and TE/TR/flip angle.*
Area of acquisition	*State whether a whole brain scan was used OR define the area of acquisition, describing how the region was determined.*

Diffusion MRI ☐ Used ☐ Not used

Preprocessing

Preprocessing software — *Provide detail on software version and revision number and on specific parameters (model/functions, brain extraction, segmentation, smoothing kernel size, etc.).*

Normalization — *If data were normalized/standardized, describe the approach(es): specify linear or non-linear and define image types used for transformation OR indicate that data were not normalized and explain rationale for lack of normalization.*

Normalization template — *Describe the template used for normalization/transformation, specifying subject space or group standardized space (e.g. original Talairach, MNI305, ICBM152) OR indicate that the data were not normalized.*

Noise and artifact removal — *Describe your procedure(s) for artifact and structured noise removal, specifying motion parameters, tissue signals and physiological signals (heart rate, respiration).*

Volume censoring — *Define your software and/or method and criteria for volume censoring, and state the extent of such censoring.*

Statistical modeling & inference

Model type and settings — *Specify type (mass univariate, multivariate, RSA, predictive, etc.) and describe essential details of the model at the first and second levels (e.g. fixed, random or mixed effects; drift or auto-correlation).*

Effect(s) tested — *Define precise effect in terms of the task or stimulus conditions instead of psychological concepts and indicate whether ANOVA or factorial designs were used.*

Specify type of analysis: ☐ Whole brain ☐ ROI-based ☐ Both

Statistic type for inference (See Eklund et al. 2016) — *Specify voxel-wise or cluster-wise and report all relevant parameters for cluster-wise methods.*

Correction — *Describe the type of correction and how it is obtained for multiple comparisons (e.g. FWE, FDR, permutation or Monte Carlo).*

Models & analysis

n/a	Involved in the study
☐	☐ Functional and/or effective connectivity
☐	☐ Graph analysis
☐	☐ Multivariate modeling or predictive analysis

Functional and/or effective connectivity — *Report the measures of dependence used and the model details (e.g. Pearson correlation, partial correlation, mutual information).*

Graph analysis — *Report the dependent variable and connectivity measure, specifying weighted graph or binarized graph, subject- or group-level, and the global and/or node summaries used (e.g. clustering coefficient, efficiency, etc.).*

Multivariate modeling and predictive analysis — *Specify independent variables, features extraction and dimension reduction, model, training and evaluation metrics.*
